# Statins and Inflammation: New Therapeutic Opportunities in Psychiatry

**DOI:** 10.3389/fpsyt.2019.00103

**Published:** 2019-03-05

**Authors:** Sung-Wan Kim, Hee-Ju Kang, Min Jhon, Ju-Wan Kim, Ju-Yeon Lee, Adam J. Walker, Bruno Agustini, Jae-Min Kim, Michael Berk

**Affiliations:** ^1^Department of Psychiatry, Chonnam National University Medical School, Gwangju, South Korea; ^2^IMPACT Strategic Research Centre, School of Medicine, Deakin University, Geelong, VIC, Australia; ^3^Department of Psychiatry, University of Melbourne, Parkville, VIC, Australia; ^4^The Florey Institute for Neuroscience and Mental Health, University of Melbourne, Parkville, VIC, Australia; ^5^Orygen, The National Centre of Excellence in Youth Mental Health, Parkville, VIC, Australia

**Keywords:** statin, depression, schizophrenia, dementia, inflammation

## Abstract

Statins, which are widely used to treat hypercholesterolemia, have anti-inflammatory and anti-oxidant effects. These are thought to be responsible for the potential effects of statins on various psychiatric disorders. In this study, we comprehensively review the literature to investigate the effects of statins on various psychiatric disorders including depression, schizophrenia, and dementia. In addition, we review adverse effects and drug interactions of statins to give clinically useful information guiding statin use in the psychiatric field. Statins seem useful in reducing depression, particularly in patients with physical disorders such as cardiovascular disease. In patients with schizophrenia, negative symptoms may be reduced by adjuvant statin therapy. Studies on cohorts at risk for dementia have generally shown protective effects of statins, while those on treatment for dementia show inconsistent results. In conclusion, statins used in combination with conventional psychotropic medications may be effective for various psychiatric disorders including depression, schizophrenia, and dementia. Further study is required to determine optimal doses and duration of statin use for the treatment of psychiatric disorders.

## Introduction

Statins (3-hydroxy-3-methylglutaryl coenzyme A (HMG-CoA) reductase inhibitors) are widely used to prevent cardiac and cerebrovascular events by treating hypercholesterolemia. Statins also have anti-inflammatory effects, including reducing C-reactive protein (CRP) concentrations (1). The effects of lowering low-density lipoprotein (LDL) cholesterol with statins may lead to anti-inflammatory actions because LDL cholesterol itself strongly promotes inflammation (2). Furthermore, statins reduce tumor necrosis factor alpha (TNF-α) and interferon gamma (IFNγ) production in stimulated T-lymphocytes, and inhibit the T helper cell (Th-1) immune response (3). Addition of statins to human hepatocytes reduces the levels of C-reactive protein induced by circulating interleukin 6 (IL-6), suggesting that the anti-inflammatory effects of statins are hepatic in nature (4). These anti-inflammatory and anti-oxidant effects of statins are potential mechanisms for the effects of statins on various psychiatric disorders.

Many kinds of statins have been approved for treatment of hypercholesterolemia. Statins can be broadly classified as lipophilic or hydrophilic, which affect their ability to permeate the brain (5). Hydrophilic statins, such as pravastatin, rosuvastatin, and fluvastatin are not able to easily cross the blood brain barrier (BBB), and are also less efficient at permeating cell membranes. Conversely, lipophilic statins, such as simvastatin, lovastatin, pitavastatin, and atorvastatin (6) are more likely to cross the BBB. Moreover, lipophilic statins enter cells via passive diffusion and are thus widely distributed in various tissues, whereas hydrophilic statins are more liver-specific. Cellular uptake features a variety of carrier-mediated mechanisms (5, 7). These distinct characteristics of hydrophilic and lipophilic statins may lead to differential effects of statins in terms of efficacy, or could lead to neuropsychiatric adverse events. What remains unclear is if the beneficial effects of statins require brain penetrance, or are mediated by peripheral or hepatic suppression of circulating cytokines, as is best evidenced for rosuvastatin (8), or especially in the elderly are predicated by vascular improvements in domains such as plaque stability and vessel inflammation (9).

Most psychotropic medications currently used in depression and schizophrenia act on monoamine neurotransmitters. However, certain proportions of patients with depression and schizophrenia do not respond to the conventional medications currently available. Curiously, patients with higher levels of peripheral cytokines may be less likely to respond to antidepressants (10). Therefore, clinicians require other medications with different mechanisms. Growing evidence indicates that inflammation is a key mechanisms of pathogenesis in many psychiatric disorders including depression, schizophrenia, and neurocognitive disorders. In addition, medications that act on inflammation have shown potential as alternative treatment methods. Therefore, many researchers have measured the effects of statins on these psychiatric disorders. In this study, we comprehensively reviewed clinical trials and epidemiological studies to investigate the effects of statins on various psychiatric disorders including depression, psychosis, and dementia. In addition, this study aimed to give clinical information on the implications for statin for use in clinical psychiatry.

## Mechanisms and Medical Comorbidity

The “classic” mechanism of action of statins involves the reduction of endogenous cholesterol biosynthesis via the inhibition of HMG-CoA reductase, a rate limiting enzyme integral to the mevalonate pathway. The corresponding reduction in hepatic cholesterol synthesis instigates translocation of membrane-bound sterol regulatory element-binding proteins to the nucleus, subsequent upregulation of LDL receptors on the surface of hepatocytes, leading to elevated clearance of LDL cholesterol from the blood (11, 12). These effects make statins effective for treating hypercholesterolemia. However, brain cholesterol metabolism is largely independent of dietary lipid intake because of the BBB. Brain cholesterol is synthesized in the central nervous system (CNS), unlike peripheral cholesterol (13). Not all statins are equally effective in terms of lowering brain cholesterol levels (14).

Interestingly, statins also have a range of so-called “pleiotropic” effects (e.g., suppressing inflammation, reducing oxidative stress, reducing T-cell activation) that have implications for extrahepatic systems including the cardiovascular system, the CNS, and the immune system (15–17). Statins are thought to exert many of these pleiotropic effects by suppressing the downstream synthesis of molecules in the mevalonate pathway, mediated through the inhibition of small GTPase prenylation and thus, isoprenoid production. Importantly, such small GTPases play essential roles in regulating a number of signaling pathways and cellular processes which are dependent on isoprenylation (18, 19). For example Ras plays a key role in cellular growth and proliferation; Rac in reactive oxygen species generation; and Rho in the proinflammatory cytokines. This inhibition of isoprenoids leads to a host of anti-inflammatory, immunomodulatory, anti-oxidative, and anti-atherosclerotic effects, including but not limited to: downregulation of transcription factors (e.g., Nuclear Factor-κB, Activator Protein-1), reduced expression of SOCS3 and CD40, suppression of cytokine (IL-1β, TNFα, IL-6), chemokine (IL-8, Monocyte Chemotactic Protein-1) and CRP production, attenuated induction of adhesion molecules (P-selectin, Very Late Antigen-4, Intercellular Adhesion Molecule-1), suppression of IFN-γ dependent co-stimulation of MHC Class II expression, as well as downregulation of T cell and monocyte activation.

Statins also reduce negative regulation of nitric oxide, lower NADPH oxidase and superoxide formation, and increasing oxygen free radical scavenging, decrease inflammatory cell infiltration, macrophage accumulation, reduce metalloproteinase activity, and expression, and attenuate activation of the NLRP3 Inflammasome (18–23). Notably, evidence also suggests statins facilitate PI3K-Akt signaling (24, 25), and crosstalk with peroxisome proliferator-activated receptor (PPAR) signaling (26) Collectively, these resultant cardioprotective, immunoprotective, and neuroprotective benefits of the aforementioned pleiotripic effects make statins worthy of investigation for treating neuropsychiatric disorders with diffuse etiologies.

Several neuroimaging studies have explored the effects of brain statins. Serial volumetric magnetic resonance imaging of patients with multiple sclerosis, a chronic inflammatory/ neurodegenerative disorder, revealed significantly less whole-brain atrophy in a high-dose (80 mg daily) simvastatin group than in a placebo group (27). Studies using positron emission tomography or diffusion tensor imaging to evaluate dementia patients have yielded conflicting results in terms of the effects of statins on neurodegeneration and white matter integrity (28). Further research is required to explore whether statins control brain atrophy and functional connectivity.

Psychiatric disorders are often associated with several somatic consequences, including hypertension, heart disease, stroke, cancer, obesity, diabetes mellitus, and osteoporosis (29). It is known that individuals with psychiatric disorders tend to have unhealthier lifestyle habits, such as drinking excessive amounts of alcohol, are more likely to smoke, eat an unhealthy diet and be more physically inactive than their peers, be less compliant with medication regimens and have poorer self-care (30). All these factors significantly contribute to the development and maintenance of the above-mentioned comorbidities.

Also, shared immunometabolic pathways have been implicated in the link between psychiatric and physical disease. Psychiatric conditions are consistently linked to disruptions in the body's stress response system (mainly the Autonomic Nervous System (ANS) and the Hypothalamic-Pituitary-Adrenal (HPA) axis (31) and are often associated with a pro-inflammatory profile (32). The chronicity of these processes might lead to several somatic consequences, including elevated blood pressure, abdominal obesity, dyslipidaemia and increased blood glucose. These conditions constitute important risk factors for cardiovascular disease (CVD), diabetes (33), cognitive impairment and even cancer (34), among others.

A recent review suggests that abdominal obesity and lipid disturbances are one of the driving forces behind the relationship between psychiatric disorders, in this case depression, and inflammation (35). Abdominal obesity gives rise to multiple immunometabolic dysregulations. White adipose tissue, especially in the abdominal area, plays as an active endocrine organ producing inflammatory cytokines and hormones (especially leptin) that disrupt important immunometabolic pathways (36). Increased leptin is a risk pathway for depression (37). Increased inflammatory activity interferes with HPA axis regulation, altering cortisol secretion and feedback, leading to a progressive feedforward loop of inflammation-related conditions (38).

Statins, with their lipid lowering, immunomodulatory, anti-inflammatory, and antioxidant properties, may act to slow or indeed prevent some of these alterations, potentially leading to interruption of the neuroprogressive cascade in these disorders (39) and decreased morbidity and mortality for individuals with psychiatric conditions. Notably, the main cause of death in psychiatric disorders remains CVD (40), where statins have its most definitive proved role (41). The possible therapeutic benefits of statins in each psychiatric disorder are described individually in this paper.

## Statins for Depressive Disorder

The pathogenesis of depression is both complex and heterogeneous. Inflammation and immune dysfunction are major contributors to the development of depression (42, 43). Inflammatory markers have been associated both with the prognosis of depression (44) and the risks of associated cardiac events and cancer (45). Peripheral pro-inflammatory cytokine signals are transmitted to the CNS via both humoral and neural pathways and may trigger depression by increasing oxidative stress (46); interacting with the hypothalamic–pituitary–adrenal axis; causing impairments in neurotransmitter systems involving glutamate, serotonin, dopamine, and noradrenaline (47–49); disrupting mitochondrial biogenesis (50); decreasing neurogenesis (51); and causing persistent and detrimental changes in the brain.

The monoaminergic theory of depression has failed to deliver novel therapeutic agents. Drugs with anti-inflammatory properties are important potential alternatives for the treatment of depression (52). Antidepressant effects of anti-inflammatory agents were noted in earlier epidemiology studies and clinical trials, including celecoxib, pioglitazone, N-acetylcysteine and statins. Besides their lipid-lowering properties, statins possess direct anti-inflammatory effects as noted above (53), which led researchers to investigate the potential impact of statins on depression. The present study summarizes previous studies focusing on the prospective association between statins and depression in Table 1.

**Table 1:** Studies investigating the associations between statin use and depression.

**References**	**Study design**	**Subjects number (mean age)**	**F/U duration**	**Assessment**	**Diagnosis**	**Statin name and dosage**	**Main findings**
**EPIDEMIOLOGICAL STUDY**							
Young-Xu et al. (54)	Prospective	606 CAD patients (67 yrs)	4–7 years	Kellner Symptom Questionnaire	Psychological well-being	Any use	Reduced risk of abnormal depression scores in CAD patients: aOR (95% CI) = 0.63 (0.43–0.93)
Stafford et al. (52)	Prospective	193 heart disease patients (64 yrs)	9 months	3M-MINI 9M-HADS	Major and minor depression dysthymia	Atorvastain, Pravastatin, Simvastatin	Reduced risk of dysthymia, minor depression, or major depression both at 3 and 9 months 3 mo: aOR (95% CI) = 0.31 (0.10–0.97) 9 mo: aOR (95% CI) = 0.21 (0.05–0.88)
Otte et al. (55)	Prospective	965 CAD patients (67 ± 11 yrs)	6 years	PHQ	PHQ ≥10 depression	Any use	Reduced risk of depression aOR (95% CI) = 0.62 (0.41–0.95)
Yang et al. (56)	Retrospective	458 depression and 1,830 controls (55 ± 9 yrs)	NA	Recorded diagnosis of depression	Depression Suicidal behavior	Any use	Reduced risk of depression aOR (95% CI) = 0.4 (0.2–0.9)
Pasco et al. (57)	Retrospective	22 Women with MDD and 323 Control (72 ± 9 yrs)	10 years	SCID using DSM-IV	MDD	Not reported	Reduced risk for MDD. Hazard ratio [HR] (95% CI) = 0.20 (0.04–0.85)
Al Badarin et al. (58)	Prospective	3,050 statin user and 625 statin nonuser (58 ± 12 yrs)	1 year	PHQ-8	PHQ-8≥10 depression	NA	No significant association in myocardial infarction patients
Redlich et al. (59)	Retrospective	4,607,990 National register data (63 ± 17 yrs)	2 years	ICD-10 codes F30–F39	Depressive disorder	Any use	Lipophilic agents- reduction in risk aOR(95% CI) = 0.92 (0.88–0.96) Hydrophilic agents-no association aOR(95% CI) = 0.85 (0.70–1.03)
Chuang et al. (60)	Retrospective (health insurance)	26,852 hyperlipidemia patients (52 ± 14 yrs)	NA	Insurance claim	Depressive disorder	NA	Reduced risk of depression in hyperlipidemia patients; HR (95% CI) = 0.81 (0.69–0.96)
Kim et al. (61)	Prospective	423 stroke patients (65 ± 10 yrs)	1 year	MINI (DSM-IV) HDRS	Major or minor depression	Any use	Reduced risk of depression in stroke patients; aOR (95% CI) = 0.54 (0.49–0.87)
Kang et al. (62)	Retrospective (health insurance)	11,218 stroke patients (71 ± 12 yrs)	1 year	ICD-9-CM 296.2, 296.3, 300.4, or 311	Depression	NA	Increased risk of depressive disorder following stroke: aHR(95% CI) = 1.65 (1.34–2.03)
Glaus et al. (63)	Prospective cohort	1,631 community population (52 ± 9 yrs)	5.2 ± 1.2 years	DIGS	DSM-IV MDD	NA	No association of MDD risk aHR(95% CI) = 1.25(0.73–2.14)
Kohler et al. (64)	Prospective (Danish Civil Registration)	872,216 SSRI users (48 [33-68] yrs)	3 years	ICD-10 F32 or F33; suicide attempt	NA	Any use	Adjunctive stain use with SSRI -Reduced risk for psychiatric hospital contacts due to depression: aHR(95% CI) = 0.64 (0.55–0.75)
**INTERVENTIONAL STUDY**							
Ghanizadeh et al. (65)	DB RCT	68 statin use and placebo (32 ± 10 yrs)	6 weeks	HDRS	DSM-IV MDD, (HDRS > 17)	Fluoxetine up to 40 mg/d + Lovastatin 30 mg/d	“Lovastatin + fluoxetine” improved depressive symptoms HDRS reduction 12.8(6.3) vs. 8.2(4.0), *P* < 0.001 No adverse effect
Haghighi et al. (66)	DB RCT	60 statin use and Placebo (32 ± 8 yrs)	12 weeks	HDRS	DSM-5 MDD, (HDRS ≥ 25)	Citalopram 40 mg/d + Atorvastatin 20 mg/d	“Atorvastatin + SSRI” improved depressive symptoms Partial remission OR 8.83(1.02–76.96): Statin user vs. non-user
Gougol et al. (67)	DB RCT	48 statin use and Placebo (35 ± 10 yrs)	6 weeks	HDRS	DSM-IV MDD, (HDRS > 17)	Fluoxetine 40 mg/d + Simvastatin 20 mg/d	“Simvastatin + fluoxetine” improved depressive symptoms No adverse effect
Abbasi et al. (68)	DB RCT	46 patients received CABG (57 ± 7 yrs)	6 weeks	HDRS	DSM-IV MDD, (HDRS ≥ 19)	Simvastatin 20 mg/d Atorvastatin 20 mg/d	Earlier and superior effect of improving depressive symptoms; simvastatin > atorvastatin No serious adverse effect
Kim et al. (7)	Naturalistic prospective observation study	446 patients with CAD (59 ± 11 yrs)	24 weeks & 1 year	HDRS, BDI	MINI: MDD or minor depressive Dis	Any use lipophilic vs. hydrophilic statins	Improved depressive symptoms and response rate: Escitalopram + statin > statin > escitalopram > none Response rate: lipophilic statins > hydrophilic statins, OR (95% CI) 3.91 (1.21–12.59)

*yrs, years; aOR, Adjusted odd ratio; CAD, coronary artery disease; MINI, Mini International Neuropsychiatric Interview; HADS, Hospital Anxiety and Depression Scale; PHQ, Patients Health Questionnaire; NA, not assessed; MDD, major depressive disorder; Dis, disorder; SCID, Structured Clinical Interview for DSM; DSM-IV, Diagnostic and Statistical Manual of Mental Disorders 4th edition; ICD-9-CM, International Classification of Diseases, Ninth Revision, Clinical Modification; ICD-10, International Classification of Diseases 10th revision; HDRS, Hamilton Depression Rating Scale; DIGS, Diagnostic Interview for Genetic Studies; DSM-5, Diagnostic and Statistical Manual of Mental Disorders 5th edition; BDI, Beck Depression Inventory*.

To date, there has been one meta-analysis including seven observational studies (four cohort, two nested case-control, and one cross-sectional study), which found that statin users were less likely to develop depression than non-users (69). In addition to this meta-analysis, four prospective studies including large populations (*n* = 26,852–4,607,990) using national register data reported that statin use was associated with a reduced risk of depression (59–61, 64). Meanwhile, two prospective studies found that statin use was not associated with worsening of depression in acute myocardial infarction (AMI) patients (58) or with depression risk in a community population (*n* = 1,631) (63). Another study reported that statin use in stroke patients was associated with heightened depression risk (62). However, this study on stroke patients (62) did not adjust for significant covariates, limiting our ability to interpret the results (70). The study of AMI patients assessed changes in depression scale scores, which were at non-significant levels at baseline (58). Moreover, the community studies that reported potential beneficial effects of statins on depression (59, 60, 64) included larger numbers of participants and younger populations compared to the study that reported a detrimental effect of statins on depression (63). We hypothesize that the direction of associations may depend on the characteristics of the participants. In healthy populations without inflammatory loading, statins may not have beneficial anti-inflammatory effects. This finding is concordant with the findings of Miller and Raison, who found that levels of inflammation predicted response to infliximab; those with high levels benefitted, and those that had low levels worsened. The cholesterol-lowering effects of statins may make fragile people (e.g., the elderly) who are likely to already have low cholesterol levels vulnerable to depression via lowered serotonin levels. Therefore, the risks and benefits of statin use may depend on patient characteristics.

Statin use could be helpful in reducing the risk of depression in populations who are experiencing excess inflammation due to physical diseases such as CVD–highly comorbid across major psychiatric disorders. Additionally, statin use could be beneficial for depression prevention in populations with balanced nutrition who, therefore, have a plentiful reserve cholesterol, in whom lowering cholesterol does not impact the onset of depression. Although further research is needed to confirm the type of statins that would be most beneficial for depression prevention, lipophilic statins (including simvastatin) that have better brain penetrance may have greater protective effects against depression than hydrophilic statins (including rosuvastatin and pravastatin) (59). Equally statins that most robustly suppress peripheral inflammation, such as rosuvastatin, evidenced in the JUPITER study, may have advantages (8).

Because epidemiological studies showed that statins have beneficial effects on mood, randomized controlled trials (RCTs) have been conducted to examine the efficacy of statins in treating depression (65–67). A meta-analysis including three double-blind RCTs in subjects with depression found that adjunctive therapy with statins in addition to antidepressants could be useful for the treatment of depression without any serious adverse effects, although this finding is limited by the small number of studies and short-term follow-up periods (6–12 weeks) (71). In addition to this meta-analysis, a 6-weeks double-blind RCT of simvastatin and atorvastatin without antidepressants was conducted in depressive patients after a coronary artery bypass graft (68). Although response rates by treatment were not significantly different, simvastatin tended to improve depressive symptoms earlier and more effectively than did atorvastatin, probably because the former drug can penetrate the BBB.

A non-randomized, 1-year prospective study of depressive patients after acute coronary syndrome (ACS) demonstrated that statins were effective for the treatment of depression independently of medical status and escitalopram use. In this study, the combination of statins and escitalopram had larger effects than either drug alone. In addition, lipophilic statins showed greater potential to improve depression than hydrophilic statins (7). Further analysis of this study population (72) found that levels of pro-inflammatory cytokines, including IL-6 and IL-18, predicted subsequent depression in patients with ACS. However, the trigger effects of IL-6 and IL-18 on depression were attenuated in patients receiving statins, suggesting that the antidepressant effects of such drugs are attributable to reductions in the actions of pro-inflammatory cytokines. These recent publications suggest that statins have independent effects with regard to improving depression (7, 68), but further research is needed with larger sample sizes and well-designed randomized trials in to clarify the potential benefits of statins alone in depression treatment.

In summary, both epidemiological and interventional studies show that statins are useful in reducing depression risk in patients with physical disorders such as CVD. However, caution is warranted before prescribing statins in the general population without higher inflammation loads or in populations with poor nutritional states and low cholesterol stores, because the cholesterol-lowering effects of statins could theoretically at least increase the risk of depression in these populations. Furthermore, in depressive patients, statins have been shown to be beneficial for improving depressive symptoms when used as an adjunctive therapy to antidepressants, but the independent effects of statins are yet to be confirmed.

## Statins for Schizophrenia

Schizophrenia is a severe chronic mental disorder characterized by delusions, hallucinations, cognitive impairment, avolition, reduced emotional expression, social withdrawal, and marked functional decline (73). Immune dysfunction and inflammation have been implicated in the pathogenesis of schizophrenia by numerous epidemiological and clinical studies (74, 75). Specifically, people with schizophrenia show increased levels of pro-inflammatory cytokines, and the vulnerability-stress-inflammation model also supports the role of inflammation in schizophrenia (76). Patients with schizophrenia have elevated blood levels of IL-1β, IL-6, and transforming growth factor-β (77), and they have elevated microglia activation in the brain compared to normal controls (78). As the associations between inflammation and schizophrenia have been found repeatedly, anti-inflammatory agents including non-steroidal anti-inflammatory drugs and acetylcysteine have been used as adjunctive therapies for improving symptoms of schizophrenia (79–81).

An important consideration for understanding schizophrenia is that 50–75% of deaths among patients with schizophrenia are due to CVD, while about 33% of deaths in the general population are due to CVD (82). Metabolic syndrome and dyslipidemia are associated with second generation antipsychotics and have very high prevalence rates in patients with schizophrenia. Statins effectively manage dyslipidaemia in patients with schizophrenia (83). As statins also exert anti-inflammatory actions, they are useful in preventing cardiovascular conditions in such patients and are employed to augment schizophrenia treatment.

To date, there have been six RCTs investigating the efficacy of statins as an adjuvant treatment for schizophrenia (Table 2). Five of these RCTs had small sample sizes of 12–36 patients in each treatment arm, and short treatment durations of 6–12 weeks. Only one study (86) had a larger sample size of 65 patients in each treatment arm, and investigated Positive and Negative Syndrome Scale (PANSS) negative symptom score over 6 months. Most of the studies followed patients who were outpatients in a stable state (e.g., stable on medication for several weeks prior to baseline assessment), whereas one study included inpatients in the active phase of the disease (89). Three studies used simvastatin 40 mg, while other studies used lovastatin 20 mg, atorvastatin 20 mg, or pravastatin 40 mg.

**Table 2:** Clinical trials investigating the efficacy of statins in patients with schizophrenia.

**References**	**Study design**	**Subject number (mean age)**	**F/U duration**	**Assessment**	**Diagnosis**	**Statin Name and Dosage**	**Main findings**
**POSITIVE FINDINGS**							
Tajik-Esmaeeli et al. (84)	DB RCT	36 statin and 36 placebo (44 ± 9 yrs)	8 weeks	PANSS	DSM-IV SPR, clinically stable	Simvastatin 40 mg/d + Risperidone 4~6 mg/d	“Simvastatin + risperidone” improved PANSS total and negative score at week 8
**POSITIVE ASSOCIATION WITHOUT SIGNIFICANCE**							
Chaudhry et al. (85)	RB RCT	12 statin and 12 placebo (18~35 yrs)	12 weeks	PANSS	DSM-IV Psychotic dis., stable	Simvastatin 40 mg/d + APs	“Simvastatin + Aps” improved PANSS total score without significant difference; little effect on PANSS negative score
Deakin et al. (86)	RB RCT	130	6 months	PANSS	NA	Simvastatin 40 mg/d + APs	“Simvastatin + Aps” improved PANSS negative score at 3 and 6 months without main effects of simvastatin
Vincenzi et al. (87)	DB RCT	30 statin and 30 placebo (44 ± 12 yrs)	12 weeks	PANSS	DSM-IV SPR, outpatients	Pravastatin 40 mg/d + APs	“Pravastatin + Aps” improved PANSS positive score at week 6, but failed to remain significant at week 12
Sayyah et al. (88)	DB RCT	21 statin (35 ± 7 yrs) and 23 placebo (40 ± 10 yrs)	6 weeks	SANS	DSM-IV SPR, chronic	Atorvastatin 20 mg/d + Risperidone 6 mg/d	“Atorvastatin + risperidone” improved SANS score without significant difference (at 4 and 6 weeks, *p* = 0.074 and 0.068, respectively).
**NEGATIVE FINDINGS**							
Ghanizadeh et al. (89)	DB RCT	20 statin and 16 placebo (30 ± 8 yrs)	8 weeks	PANSS	DSM-IV SPR, inpatients	Lovastatin 20 mg/d + Risperidone 2~8 mg/d	No difference of PANSS score changes No serious adverse effect

*Yrs, years; APs, antipsychotics; DB, double-blinded; RB, rater-blinded; RCT, randomized, placebo-controlled trial; DSM-IV, Diagnostic and Statistical Manual of Mental Disorders 4th edition; PANSS, Positive and Negative Syndrome Scale, dis., disorder; SANS, Scale for the Assessment of Negative Symptoms; SPR, Schizophrenia; NA, not applicable*.

One study (84) showed that statin add-on therapy for schizophrenia patients was superior to placebo in terms of improving negative symptoms as measured by the PANSS subscale evaluating blunted affect, emotional withdrawal, apathetic social withdrawal, and poverty of speech. Although negative symptoms constitute the major barrier to functional recovery in patients with schizophrenia, the current antipsychotics exert only modest effects on negative symptoms (90, 91). Therefore, studies showing effects of statins on negative symptoms in patients with schizophrenia could have important clinical implications. Another study (89) did not show any effect of statins. The four remaining studies (85, 87, 88) reported non-significant benefits of statins. Most studies noted no significant differences in the adverse event rates between the statin user and non-user groups.

The participants of the study that reported a significant reduction in PANSS negative scores had the lowest baseline PANSS score among the six RCTs (84). This implies that the effect of statins may be more pronounced in stabilized patients than acutely ill patients. Another consideration is the type of antipsychotic medication used. There may be interactions of statins and antipsychotics, because some antipsychotics also have anti-inflammatory actions (92). Appropriate statin use may also affect the results since lipophilic statins, which can cross the BBB more readily, are more likely to interact with central brain regions (7). Simvastatin, which is the most lipophilic statin, was the most commonly used statin type in RCTs. Whether lipophilic statins improve inflammatory markers in patients with schizophrenia should be studied further.

Although there has been no study of the optimal dose and duration of statin therapy in schizophrenia, a few studies suggested the advantages of high dosage and long duration of statin therapy for CVD (93, 94). An animal study showed that hyper-locomotive activity and reduced anxiety-like behavior via NMDA receptor upregulation were initiated after high-dose simvastatin, which was higher than clinical dosages (95). Previous studies on N-acetylcysteine, which inhibits oxidative and inflammatory pathways, reported clear evidence of efficacy only after 6 months (96, 97), and a replication study noted benefits only after 9 and 12 months (98). Therefore, long-term treatment with high-dose statins may better alleviate psychotic and negative symptoms in patients with schizophrenia.

The lipid-lowering effects of statins may alleviate symptoms of schizophrenia, because studies have suggested associations between hyperlipidemia and the pathophysiology of schizophrenia (99, 100). One study found that pravastatin significantly decreased the PANSS positive subscale scores, commencing at week 6, in schizophrenia patients, but the decrease failed to remain significant to 12 weeks (87). Interestingly, the similar pattern of decrease at 6 weeks and increase at 12 weeks was found with levels of triglycerides, LDL-cholesterol, and total cholesterol. This suggests a link between lipid levels and the psychopathology of schizophrenia. However, we should consider that reduced efficacy for both psychotic symptoms and cholesterol levels could be due to poor adherence to statin medications. Additionally, a study on patients taking clozapine revealed that increases in the serum total cholesterol and triglyceride levels significantly predicted reductions in the PANSS total and/or negative subscale scores (101, 102). Furthermore, a positive longitudinal association was evident between changes in cholesterol levels and improved global cognition, particularly in verbal memory (103). Thus, further study is required to understand how changes in the serum levels of lipids and inflammatory reactions relate to changes in the symptoms of schizophrenia during statin use, and how these relationships vary with different antipsychotic drugs.

In summary, the anti-inflammatory actions of statins are expected to alleviate symptoms of schizophrenia as an augmentation to other drugs, and they have the added benefits of treating metabolic abnormalities such as hyperlipidemia to prevent CVD. We recommend the use of a sufficient dose of statins for at least 12 weeks in stable patients with schizophrenia for functional recovery as well as liberal use of statins in those with high levels of cardiovascular risk. Further studies are required in various populations and stages of illness.

## Statins for Neurocognitive Disorders

Dementia has complex and heterogenous etiologies, including cerebrovascular disease, amyloid plaques, and tauopathy (104). Alzheimer disease (AD) is the most common cause of dementia and represents one of the largest burdens of disease in elderly persons (105). Evidence suggests that the deposition of ß-amyloid plaques and inflammatory processes in the CNS play important roles in the development and progression of AD (106, 107). Excessive amyloid beta (Aβ) accumulation is a hallmark feature of the AD neurodegenerative cascade (108). The dysregulation of the Aβ clearance process is characterized by elevated levels of pro-inflammatory cytokines, and this induces Aβ accumulation and continuous immune activation (109).

Defects in brain cholesterol homeostasis have been implicated in neurodegenerative diseases including AD and cognitive deficits typical of old age (13). Most brain cholesterol is synthesized in astrocytes; ApoE shuttles cholesterol (in a lipoprotein complex) from these cells to neurons. Therefore, ApoE may play an important role in cholesterol homeostasis in aging and diseased brains (110). Furthermore, cholesterol homeostasis may significantly affect the synthesis, deposition, and clearance of β-amyloid plaques (111, 112). The major brain cholesterol metabolite 24S-hydroxycholesterol (24S-OHC) may affect the NMDA receptor, in turn triggering cell death associated with AD (113). Statins exert anti-inflammatory and cholesterol-lowering effects in the brain, and also reduce the levels of oxysterols such as 24S-OHC (114). Statins may thus be useful in preventing/managing AD. Previous research on the association between statin use and AD, derived from cardiovascular studies, suggested that elective statin use has a beneficial effect on AD (115).

Table 3 summarizes previous studies investigating the associations between statin use and AD. Epidemiological cross-sectional and case-control studies have generally found that statins usefully prevent AD (119, 120, 125). Several prospective studies on the incidence of statin use and AD have also shown a protective association, although these studies have limitations. The Adult Changes in Thought (ACT) study was a prospective study that found that statin use may be associated with reduced risk of AD, particularly in those younger than 80 (118). The 2-year follow-up of the Alzheimer's Disease Anti-inflammatory Prevention Trial (ADAPT) found reduced risk of AD in people taking statins, but it is important to note that participants regularly using NSAIDs were excluded, but non-statin lipid lowering agent use was permitted (117). Conversely, the Cache County Study found no association between statin use and the risk of AD over 72 weeks (116). There was no randomized clinical trial assessing statin use and risk of developing AD. A large primary prevention study of statins in the elderly, STAREE will explore this outcome (126).

**Table 3:** Studies investigating the associations between statin use and Alzheimer disease (AD).

**References**	**Study design**	**Subject number (mean age)**	**F/U duration**	**Assessment**	**Diagnosis**	**Statin Name and Dosage**	**Main findings**
**EPIDEMIOLOGICAL STUDY**							
Zandi et al. (116)	Prospective	4,895 (statin = 73 ± 5 yrs, nonuser = 76 ± 7 yrs)	3 years	DSM-III-R	AD	Any use	No association with subsequent onset of AD; aHR(95% CI) = 1.19 (0.35–2.96)
Sparks et al. (117)	Prospective	2,528 (75 ± 4 yrs)	5 years	DSM-IV NINCDS-ADRDA	AD	Any use	Reduced risk of AD; aHR(95% CI) = 0.33 (0.11–0.98)
Li et al. (118)	Prospective	3,099 (statin = 74 ± 6, non-user = 76 ± 6 yrs)	6 years	NINCDS-ADRDA, DSM-IV	AD	Any use	Reduced risk of AD; aHR(95% CI) = 0.62 (0.40–0.97) Strength of the statin diminished with age
Zissimopoulos et al. (119)	Retrospective	399,979 (women 76 and men 75 yrs)	5 years	ICD-9	AD	atorvastatin, pravastatin, rosuvastatin	Reduced risk of AD diagnosis for women: aHR(95% CI) = 0.85 (0.82–0.89) for men: aHR(95% CI) = 0.88 (0.83–0.93)
Zamrini et al. (120)	Case control	505 AD-paired patients (73 yrs)		ICD-9	AD	Any use	Reduced risk of AD; aOR (95% CI) = 0.61 (0.42–0.87)
**INTERVENTIONAL STUDY**							
Simons et al. (121)	RCT	44 statin and 68 placebo (68 ± 8 yrs)	26 weeks	NINCDS-ADRDA	mild-to-moderate AD	Simvastatin 80 mg	A small but significant reduction of Abeta40 in the CSF of mild but not moderate AD patients.
Sparks et al. (122)	RCT	32 statin and 31 placebo (78 ± 1 yrs)	1 year	DSM-IV NINCDS-ADRDA	mild-to-moderate AD	Atorvastatin 80 mg	Atorvastatin may slow the progression of mild to-moderate AD
Feldman et al. (123)	RCT	297 statin and 317 placebo (74 ± 8 yrs)	72 weeks	ADAS-Cog	mild-to-moderate AD	Atorvastatin 80 mg	No significant clinical benefit
Sano et al. (124)	RCT	204 statin and 202 placebo (75 ± 9 yrs)	18 months	ADAS-Cog	mild-to-moderate AD	Simvastatin 40 mg	No benefit on the progression of symptoms despite significant lowering of cholesterol.
Lin et al. (125)	Case control	719 AD-paired patients	≥1 year	ICD-9 DSM-IV	mild-to-moderate AD	Any use	Early stain use exhibited a 0.85-risk (95% CI = 0.76–0.95) to have AD progression than those without.

*Yrs, years; RCT, randomized controlled trial; DSM, Diagnostic and Statistical Manual of Mental Disorders; aHR, Adusted Hazard Ratio; NINCDS-ADRDA, National Institute of Neurological and Communicative Disorders and Stroke and the Alzheimer's Disease and Related Disorders Association; ADAS-Cog, Alzheimer's Disease Assessment Scale-Cognitive Subscale test; ICD-9, International Classification of Diseases, Ninth Revision*.

There have been four published RCTs of statins as an intervention in patients with mild to moderate AD. The Lipitor's Effect in Alzheimer's Dementia (LEADe) study was the largest with 640 patients, and found that a regimen of atorvastatin (80 mg/day) plus donepezil was not associated with significant benefits for treatment over 72 weeks (123). Similarly, a medium-sized, placebo-controlled, double-blind, randomized study found that simvastatin (20~40 mg) had no benefit for the progression of symptoms in individuals with mild to moderate AD (124). In contrast, a RCT showed that AD progressed more slowly (as measured by the Mini-Mental State Examination) in patients treated with simvastatin (80 mg/day) than placebo, over 26 weeks (121). Furthermore, this study showed that statins also decreased levels of beta-amyloid in the cerebrospinal fluid of patients with mild AD. In the 1-year follow-up of the Alzheimer's Disease Cholesterol-Lowering Treatment (ADCLT) trial study, there were also positive effects for treatment outcomes between 32 patients receiving 1 year of atorvastatin (80 mg/day) and 31 receiving placebo (122). Atorvastatin significantly improved memory performance as measured by the ADAS-Cog instrument after 6 months of treatment in patients with mild to moderate AD. These inconsistent results may be attributed by differences in sample size, statin dosage, characteristics of the statin used (lipophilic vs. hydrophilic), baseline lipid level, and assessment tools used for cognitive function.

In summary, statins may reduce the incidence of AD (126). However, RCTs assessing cognition in AD patients have yielded inconsistent results (127). A key point emerging from this research is the importance of the timing of statin treatment for achieving benefits in AD. Because AD progresses over long periods of time, future studies should include long-term follow-up periods to enable detection of any effects of statin treatment and might usefully focus early in the illness course, such as mild cognitive impairment.

## Statins for Other Psychiatric Disorders

There have been several clinical trials of statins for delirium prevention or treatment in critically ill patients. Based on the neuroinflammatory hypothesis of delirium, which is characterized by acute release of inflammatory mediators during critical illness, the pleiotropic effects of statins may prevent or attenuate delirium due to their effects on neutrophil migration, BBB injury, and inflammation (94, 128). However, a review of the literature regarding the use of statins for delirium prevention or treatment reveals no clear overall conclusions. Differential effects of statins on neuroinflammation during delirium may be due to treatment with lipophilic vs. hydrophilic statins. The current study demonstrated that the use of a hydrophilic statin (pravastatin) was associated with reduced delirium incidence compared with a lipophilic statin (atorvastatin), but the reverse has also been found (129). A recent comprehensive meta-analysis found that statins did not reduce the incidence of delirium in physically ill patients (130). There are many confounding factors that might account for these inconsistent results, including heterogeneity of study designs, variability of patient populations, the multifactorial nature of delirium, inconsistent delirium assessments, limited study power and lack of information on co-administration of other neuropsychiatric medications. Therefore, well-designed studies on delirium are still needed.

When considering bipolar disorder as a multisystemic inflammatory disease (131), it is important to examine the effect of statins on the manic phase as well as the depressive phase. An RCT evaluating lovastatin as an adjuvant to lithium in patients in the manic phase of bipolar disorder found that lovastatin neither exacerbated nor improved manic symptoms (132). That study suggested that the combination of statins with lithium is well-tolerated in patients with bipolar disorder, without evidence of exacerbation of mania by the antidepressant effects of statins.

## Adverse Events Associated With Statins

The usual doses of statins are generally safe, being rarely associated with clinically significant adverse events (133). However, clinicians should know the general adverse events when prescribing statins. Statin-associated muscle symptoms (SAMs) are clinically important side effects of statins. SAMs are the most common side effects, reported by 10–25% of patients undergoing statin therapy, and are also the most common cause of statin discontinuation (134–136). SAMs range in severity from muscle cramps and weakness to creatine kinase (CK) elevation and rhabdomyolysis. Severe muscle damage is relatively rare among SAMs, but rhabdomyolysis should be distinguished from neuroleptic malignant syndrome (NMS), which is a rare but life-threatening disease that can occur with antipsychotic medication (137). Recent studies have found that patients who experienced non-severe SAMs can tolerate statins upon blinded re-challenge (138–140). Many reported adverse events can be predicated on expectancy and nocebo phenomena (141, 142). Therefore, it is necessary to consider a comprehensive approach and management such as patient assessment, treatment according to severity, re-assessment and considering other treatment options for SAMs (143). Neuropathy is most likely to develop after long-term treatment, and it generally resolves after the discontinuation of statins (144).

Statin use increased diabetes risk by 9–13% in a meta-analysis of randomized trials (145). Additionally, high-dose statins increased the risk of diabetes compared to that associated with standard-dose statins (146). Predictors of new-onset diabetes in patients treated with atorvastatin were baseline fasting blood glucose level, body mass index, hypertension, and fasting triglyceride level (147). However, a study on diabetes risks associated with statin therapy showed that patients lacking baseline risk factors (metabolic syndrome, impaired fasting glucose level, body mass index >30 kg/m^2^, or HbA1c >6%) did not develop diabetes (148). In addition, several studies have suggested that statin-mediated prevention of cardiovascular disease should receive more emphasis than the risk of diabetes (149–151). Risk factors for diabetes should be routinely evaluated before prescribing statins. L-carnitine (500–1,000 mg twice daily) may prevent any increase in blood sugar levels (152).

Although the serum levels of hepatic transaminases may increase in patients taking statins, routine measurement of liver enzyme levels is not required (153). Other possible physical side effects of statins are hemorrhagic stroke, decreased renal function, tendon rupture, interstitial lung disease, and low testosterone levels (154).

Risk for potential neuropsychiatric adverse events may also be increased in the use of long-term high doses of statins. Mood disturbance, sleep changes, cognitive impairment, and suicide have been reported in patients taking statins, although casual links are uncertain (69, 155–157). Clinicians should assess neuropsychiatric adverse events associated with statin use.

The US Food and Drug Administration recently issued a warning stating that statins could cause mild cognitive impairment (158). Some case reports and several studies have reported small cognitive decreases in patients on statin therapy (159–161); other studies have found that statins reduced cognitive decline in older adults (117, 162, 163). A recent systematic review and meta-analysis found no significant association between statin use and cognitive impairment (164, 165). Neurocognitive impairment following the initiation of statins appears to depend on statin type; lipophilic statins are associated with more cognitive impairments (159, 166), while clinical trials using hydrophilic statins, which are less likely to cross the blood brain barrier, did not show any significant cognitive impairment.

It seems biologically plausible that lowering cholesterol levels with statins could cause several changes in the serotonergic system, which might lead to increased irritability and violent ideation (167–169). Despite some case reports of mood disturbance with statin use (170, 171), evidence of any relationship between statins and mood is conflicting. It has been suggested that statins might be associated with a higher rate of sleep disturbance, especially for lipophilic statins (172, 173). Multi-methodological approaches using different databases suggest that statin use was associated with an increased risk of sleep disturbances (including sleep initiation and maintenance) and parasomnias (173). However, there is no conclusive evidence that any particular statin is more likely to cause sleep disturbances than other statins, and a recent meta-analysis found that statins exerted no significant adverse effects on sleep duration or efficiency (155).

The literature regarding statin-associated suicide risk remains limited, and there is no proven association between statin use and increase in suicide (56). A Danish population-based study (*n* = 642,058) showed that the SSRI-statin (citalopram 57.2%, simvastatin 92%) group was associated with fewer psychiatric hospital contacts and no increase in suicidal behavior compared to populations who used SSRI alone (174). Overall, even if the cholesterol-lowering effects of statins could be associated with potential adverse events, there has been little conclusive evidence of statins causing serious side effects that would outweigh the advantages of this medication. Furthermore, the advantageous anti-inflammatory actions of statins may alleviate any potential negative effects of lower cholesterol.

## Drug Interactions of Statins

Metabolism of statins is complex, and begins with absorption, followed by hepatic uptake, metabolism, and elimination (175). Individual characteristics and multiple transporters acting on each phase interact with concomitant medications, leading to changes in serum concentrations of statins, may change. Among multiple pathways, the role of the cytochrome P-450 (CYP) system has been most commonly described (133). Most statins are metabolized by CYP 3A4. Exceptions include fluvastatin and rosuvastatin, which are largely metabolized by CYP 2C9 (176), and pravastatin, which is mainly metabolized by renal clearance (177). Antidepressants, antipsychotics, anxiolytics, and cognitive enhancers that are metabolized by CYP isoenzymes can increase serum statin concentrations by competing for catabolism and vice versa (176). Also, inhibition and enhancement of the CYP isoenzymes by psychotropic medications can increase or decrease serum concentrations of statins. One case report described a 79-year-old female prescribed nefazodone 300 mg daily for 8 years, who then took simvastatin 40 mg daily, and subsequently developed rhabdomyolysis, perhaps attributable to potent inhibition of CYP 3A4 by nefazodone (178). CYP status must be evaluated when combining antidepressants and statins (178).

Drug interactions between statins and antipsychotics may influence the efficacy of antipsychotics through P-glycoprotein 1 (P-gp), which is a transporter in the BBB and regulates brain tissue for access of centrally acting drugs. Both statins and some antipsychotic drugs are substrates of P-gp (179). Thus, statins and antipsychotic agents may act synergistically in terms of CNS access (180). The increased CNS levels may improve psychotic symptoms in schizophrenia patients (180).

## Discussion and Conclusions

The degree of lipophilicity required to cross the BBB may be associated with direct effects of statins on the brain, as might their capacity to suppress peripheral cytokines. Neuropsychiatric side effects of statins including neuropathy and cognitive impairment may be more closely associated with the lipophilic statins, probably because they are more likely to cross the BBB. However, lipophilic statins such as simvastatin have demonstrated effectiveness for improving depression as well as the negative symptoms of schizophrenia. Clinicians should be aware of these contradictory findings that lipophilic statins are associated with a higher potential for neuropsychiatric adverse events as well as increased efficacy for psychiatric symptoms when selecting the type of statin to prescribe.

The half-life of cholesterol in the adult brain ranges from 6 months to 5 years; in contrast, the half-life of plasma cholesterol is only a few days. Thus, any brain lipid-lowering effect of statins may be very slow (13, 181). Statins exert anti-inflammatory and anti-oxidant effects, which may explain their benefits in patients with various psychiatric disorders. Therefore, for psychiatric patients, high-dose long-term statin therapy may be required. Future long-term studies should explore the effects of statins in various psychiatric disorders.

In conclusion, statin therapy appears to be safe in the majority of patients, and the benefits of statin use far outweigh the potential risks. Statins used with conventional psychotropic medications may be effective in various psychiatric disorders including depression, schizophrenia, and the risk for dementia. Statins seem useful in reducing depression, particularly in patients with physical disorders. In patients with schizophrenia, negative symptoms may be reduced by adjuvant statin therapy. Studies on cohorts at risk for dementia have generally shown protective effects of statins, while those on treatment for dementia show inconsistent results. Further study is required to investigate optimal statin dose and duration of use. In addition, population studies using statins are ideal candidates for further investigations of the efficacy of statins in mitigating the risk and prevention of psychiatric conditions and their cardiovascular comorbidities.

## Author Contributions

SWK, JMK, and MB designed the strategy for the present review. SWK, HJK, MJ, JWK, JYL, AW, and BA wrote the first outlined of the review. SWK, JMK, and MB critically revised the draft. All authors read and approved the submitted version.

### Conflict of Interest Statement

The authors declare that the research was conducted in the absence of any commercial or financial relationships that could be construed as a potential conflict of interest.
